# Understanding developmental plasticity as adaptation requires an inter-generational perspective

**DOI:** 10.1093/emph/eox023

**Published:** 2018-02-05

**Authors:** Jonathan C K Wells

**Affiliations:** Childhood Nutrition Research Centre, Population, Policy and Practice Programme, UCL Great Ormond Street Institute of Child Health, 30 Guilford Street, London WC1N 1EH, UK

**Keywords:** developmental plasticity, adaptation, parental effects, parent-offspring conflict

## Abstract

In this issue of Evolution, Medicine and Public Health, Lea and colleagues argue that there are major advantages to bringing together biomedical and evolutionary perspectives on plasticity. To develop this approach, they propose two contrasting scenarios for ‘developmental plasticity as adaptation’: that it reflects adjustments to resolve the effects of early ‘constraints’, or that it adjusts phenotype to ecological cues in anticipation of similar conditions in adulthood. Yet neither scenario highlights the unique role of maternal phenotype, mediated by maternal investment strategy, in generating such constraints or cues. Developmental plasticity is greatest during the period when all ecological influences on the offspring are transduced by maternal phenotype. If the offspring adapts during this period, then the target of that adaptation is to maternal phenotype. Ignoring the inter-generational source of early constraints or cues prevents development of a comprehensive adaptive framework, because developmental plasticity is fundamentally relevant to the fitness of both offspring and parents.

Lea *et al.*. have produced a clear and thoughtful review of developmental plasticity as adaptation [1]. Paradoxically, while I agree with their aims and with many of their individual points, I have concerns about their overall perspective. I offer some critical comments, aimed at drawing greater attention to the need to address parental phenotype in both evolutionary and biomedical perspectives on plasticity.

Regarding ‘plasticity as adaptation’, Lea *et al.* discern two contrasting scenarios: that it reflects adjustments to resolve the effects of early ‘constraints’ or that it adjusts phenotype to ecological cues in anticipation of similar conditions in adulthood. Surprisingly, however, they place minimal emphasis on the initial *source* of both ‘constraints’ and ‘predictive cues’. Crucially, the primary period of mammalian developmental plasticity falls within the period of maternal physiological care (pregnancy and lactation). Ignoring the inter-generational source of early constraints or cues prevents development of a comprehensive adaptive framework, because developmental plasticity is fundamentally relevant to the fitness of both offspring and parents [2–5].

Before biomedical researchers formulated the ‘developmental origins of adult health and disease’ hypothesis, zoologists had already identified the importance of ‘parental effects’, namely the capacity of parents to influence the phenotype of their offspring beyond direct genetic transmission [6]. Parental effects can be studied across a huge range of species, and this reminds us of their importance when we try to link evolutionary and biomedical perspectives on plasticity in humans.

In mammals, mothers exert phenotypic effects through several physiological pathways, whilst fathers can do so through imprinting of the sperm. Importantly, however, mothers also have substantial capacity to *buffer* the offspring from external ecological effects [2–4, 7, 8]. In consequence, the primary ecological factor to which the fetus and infant are exposed is maternal phenotype, with paternal effects modulating that relationship. If plasticity during fetal life represents adaptation, that adaptation is primarily to maternal phenotype rather than to the external environment, since *there is no ecological stress that is not mediated by maternal phenotype* [7, 8]. The same scenario applies to some extent during lactation, though the offspring is now exposed to some ecological factors directly (e.g. the thermal environment; pathogens; non-maternal sources of nutrition).

One could therefore redefine both early developmental plasticity and parental effects in a unified framework. During pregnancy, for example one could define (a) a maternal non-genetic effect as anything that elicits a plastic response in the fetus and (b) fetal plasticity as the consequence of maternal non-genetic effects.

As Trivers and Haig have emphasized [9, 10], any ‘unit’ of maternal investment has non-identical implications for parental and offspring fitness. We can think of parents and offspring participating in two interacting dynamic games, where, for example mothers seek to maximize fitness by investing across competing offspring, and each individual offspring adjusts its allocation of investment across competing life history functions to maximize its own fitness [4]. Feedback between these games alters the optimal strategy within each of them, hence offspring plasticity is fundamentally related to parental fitness. Birth order provides a valuable example of an early life exposure that shapes long-term phenotype and life history trajectory, but where the variability in maternal investment is neither indicative of an external constraint nor can it offer predictive cues of the future adult environment. Birth order is thus ‘useless information’ [8], and yet it elicits plastic responses in the offspring that can only be understood in the context of parental fitness.

Given extensive evidence of the ability of human mothers to gestate fetuses even in famine conditions, it is surprising that maternal buffering—the ‘withholding’ of ecological information’—is still given little emphasis in most discussions of the adaptive nature of developmental plasticity. In the Dutch Hunger Winter, maternal energy intake declined by 50–60%, and yet the reduction in birth weight averaged only ∼9% [8]. What the fetus is directly exposed to is maternal homeostatic capacity and various forms of capital, which can substantially suppress external ecological stresses. Previously, I suggested that the *duration* of early plasticity is reciprocally related to the duration of maternal care: sensitive periods are obliged to close when the developing offspring is no longer protected by maternal buffering [4].

Whilst early exposure to maternal capital offers major benefits to the offspring, it can also potentially generate costs, for maternal health and social rank emerge as key determinants of offspring plastic responses. Mothers with physiological conditions that perturb the capacity for homoeostasis (infection, obesity, hypertension, and gestational diabetes) transmit detrimental metabolic effects to their offspring [3, 8]. Low maternal rank, which can only be understood in terms of the population social hierarchy, can greatly diminish the opportunity for nutritional investment [7, 8]. Yet the majority of accounts of developmental plasticity as adaptation, on which Lea *et al.* have based their review, consider parents merely as passive vehicles for transmitting external ecological information.

After the period of parental care has ended, developmental trajectory may still demonstrate elements of plasticity elicited directly by the external environment. But the traits that are plastic from childhood onwards are typically different from those that are plastic earlier. Further, later plastic responses occur in the context of the earlier responses to parental phenotype. Again, these points are essential to address for both biomedical and evolutionary perspectives on developmental plasticity.

I fully agree with Lea and colleagues that there are major advantages to bringing together biomedical and evolutionary perspectives on plasticity, but without considering parents as the primary cause of initial plastic responses in the offspring generation, I do not believe an accurate perspective on ‘developmental plasticity as adaptation’ is actually possible.

**Conflict of interest:** None declared.
